# Extracapsular Hepatocellular Adenoma: A Diagnostic Dilemma

**DOI:** 10.7759/cureus.8928

**Published:** 2020-06-30

**Authors:** Hiffsa Taj, Isin Comba, Sundeep Kumar, Bhatia Lakhinder

**Affiliations:** 1 Internal Medicine, University of Central Florida College of Medicine, Orlando, USA; 2 Cardiovascular Disease, Saint Louis University Hospital, St. Louis, USA; 3 Gastroenterology, Osceola Regional Medical Centre, Orlando, USA

**Keywords:** hepatocellular adenoma, liver mass, liver, kehr's sign, extracapsular adenoma

## Abstract

Hepatocellular adenoma (HCA) is a benign neoplasm of the hepatic parenchyma. The use of oral contraceptives (OCP) in women is the most well-established risk for the development of HCA. HCA commonly presents as an intracapsular mass of the liver but there are very few cases of extracapsular HCA. This is a case of a middle-aged female who presented to the emergency department with left shoulder pain and epigastric tenderness on physical exam. Subsequent imaging of the abdomen revealed a mass arising from the anterior wall of the stomach, with evidence of surrounding hemorrhage. The patient underwent exploratory laparotomy that revealed free blood in the peritoneum and a hemorrhagic mass arising from the stomach wall. The mass was successfully removed with no postoperative complications. Histopathological examination of the mass was consistent with an infarcted inflammatory HCA. This case illustrates this unusual presentation of a rare diagnosis.

## Introduction

A hepatocellular adenoma (HCA) is a benign proliferation of mature hepatocytes and consists 2% of all liver neoplasms [1,2]. Its estimated incidence is 1/1,000,000 per year in the general population and 3-4 per 100,000 in women on long-term oral contraceptives (OCP) as opposed to 0.13 per 100,000 in non-OCP-users [3]. HCA is almost always intracapsular and cases originating from an ectopic liver are extremely rare with three other cases reported in the English literature. Herein, we report a unique case of complicated extracapsular HCA presenting with left shoulder pain. 

## Case presentation

A 43-year-old woman presented to the emergency department with a two-day history of left shoulder pain. The patient described the shoulder pain as sharp, constant, and non-radiating. She did not have any known past medical or surgical histories, and her only medication was OCP pills. On review of systems, she reported epigastric discomfort without nausea, vomiting, lack of appetite or recent weight changes. On physical exam, the patient was tachycardic with otherwise stable vital signs. Abdominal exam revealed epigastric tenderness with no guarding or rebound. Initial laboratory work is depicted in Table 1.

**Table 1:** Initial lab work on admission.

Lab	Result	Lab range
White cell count	10.31	4-12 K/mm3
Red cell count	4.04	4-5.2 M/mm3
Hemoglobin	12.6	12-16 gm/dL
Hematocrit	35.7	37-47%
Platelet count	347	130-400 K/mm3
Sodium	138	136-145 mmol/L
Potassium	4.2	3.7-5.1 mmol/L
Chloride	104	98-107 mmol/L
Carbon Dioxide	25	21-32 mmol/L
Blood urea nitrogen (BUN)	19	7-18 mg/dL
Creatinine	0.78	0.55-1.3 mg/dL
Glucose	136	74-106 mg/dL
Calcium	8.7	8.4-10.1 mg/dL
Total bilirubin	0.8	0.2-1.5 mg/dL
Aspartate aminotransferase (AST)	58	10-37 IU/L
Alanine aminotransferase (ALT)	58	12-78 IU/L
Alkaline phosphatase	42	45-117 IU/L
Total protein	7.6	6.4-8.2 g/dL
Albumin	3.8	3.4-5.0 g/dL
Lipase	114	73-393 unit/L

**Figure 1:**
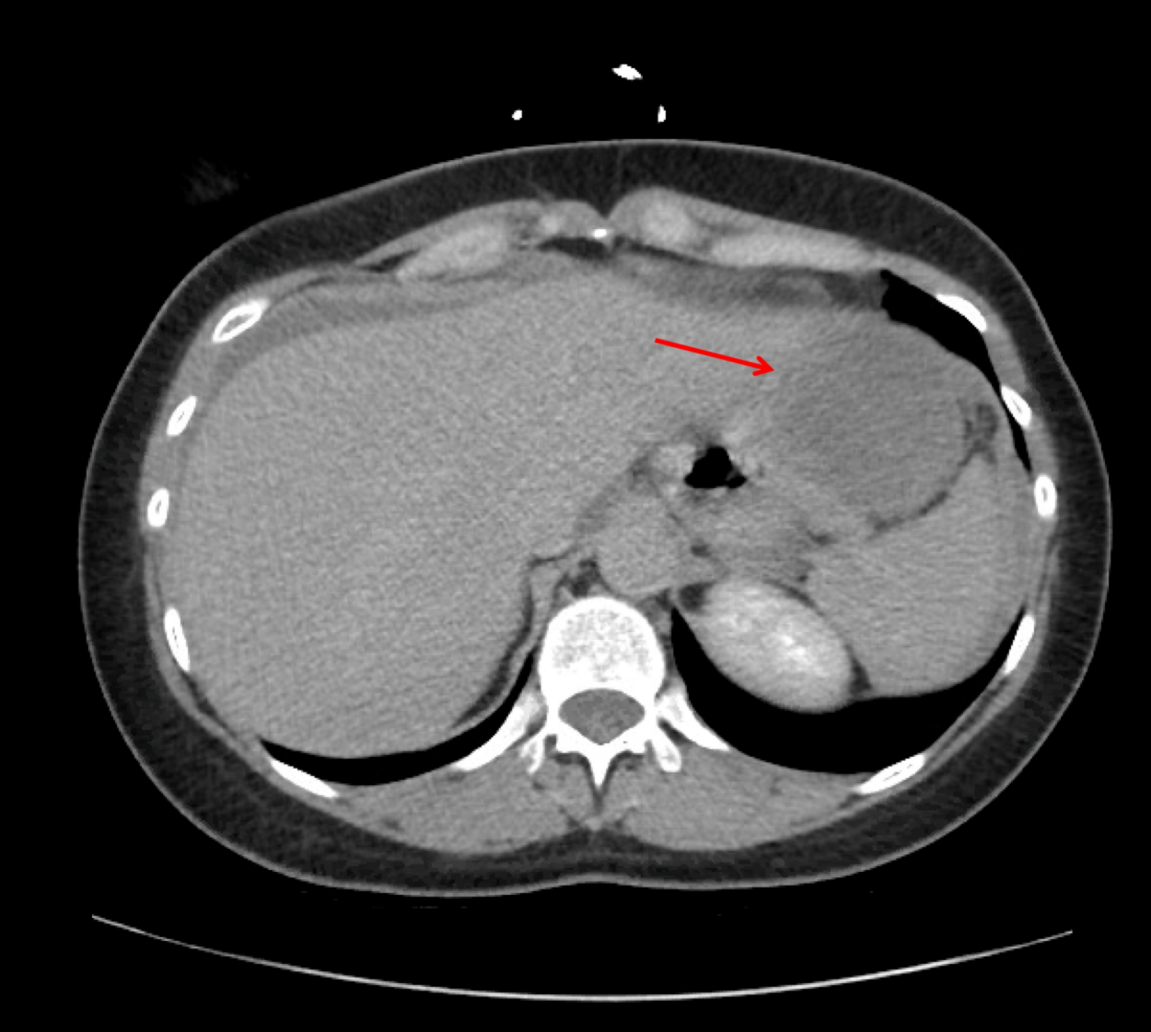
Contrast-enhanced CT abdomen (axial section) showing an irregular heterogeneous mass arising from the anterior wall of the stomach.

Initial computed tomography (CT) scan of the abdomen revealed a large heterogeneously enhancing mass arising from the anterior wall of the stomach with a small amount of free fluid surrounding the liver and a moderate amount of fluid in the pelvis (Figure 1).

**Figure 2:**
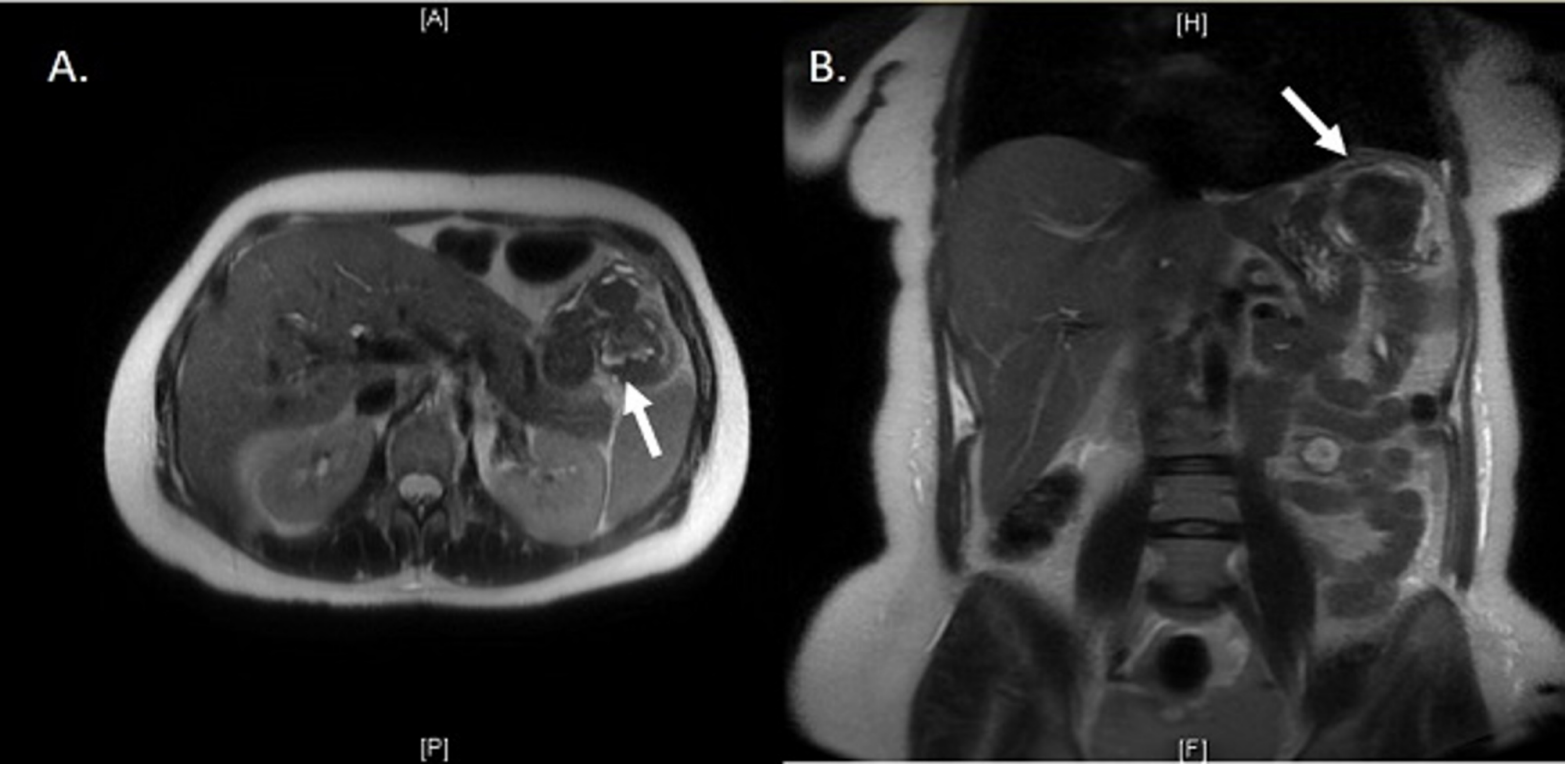
T2 weighted MRI of the abdomen demonstrated a hypoenhancing mass measuring 4 x 7.2 x 8 cm in maximum anteroposterior, transverse, and craniocaudal dimensions (arrow). (A) transverse view, (B) coronal view.

Contrast-enhanced magnetic resonance imaging (MRI) of the abdomen demonstrated an hypoenhancing exophytic mass from the anterior wall of the stomach with evidence of small volume internal and adjacent hemorrhage. The mass measured 4 x 7.2 x 8 cm in maximum anteroposterior, transverse, and craniocaudal dimensions (Figure 2).

**Figure 3:**
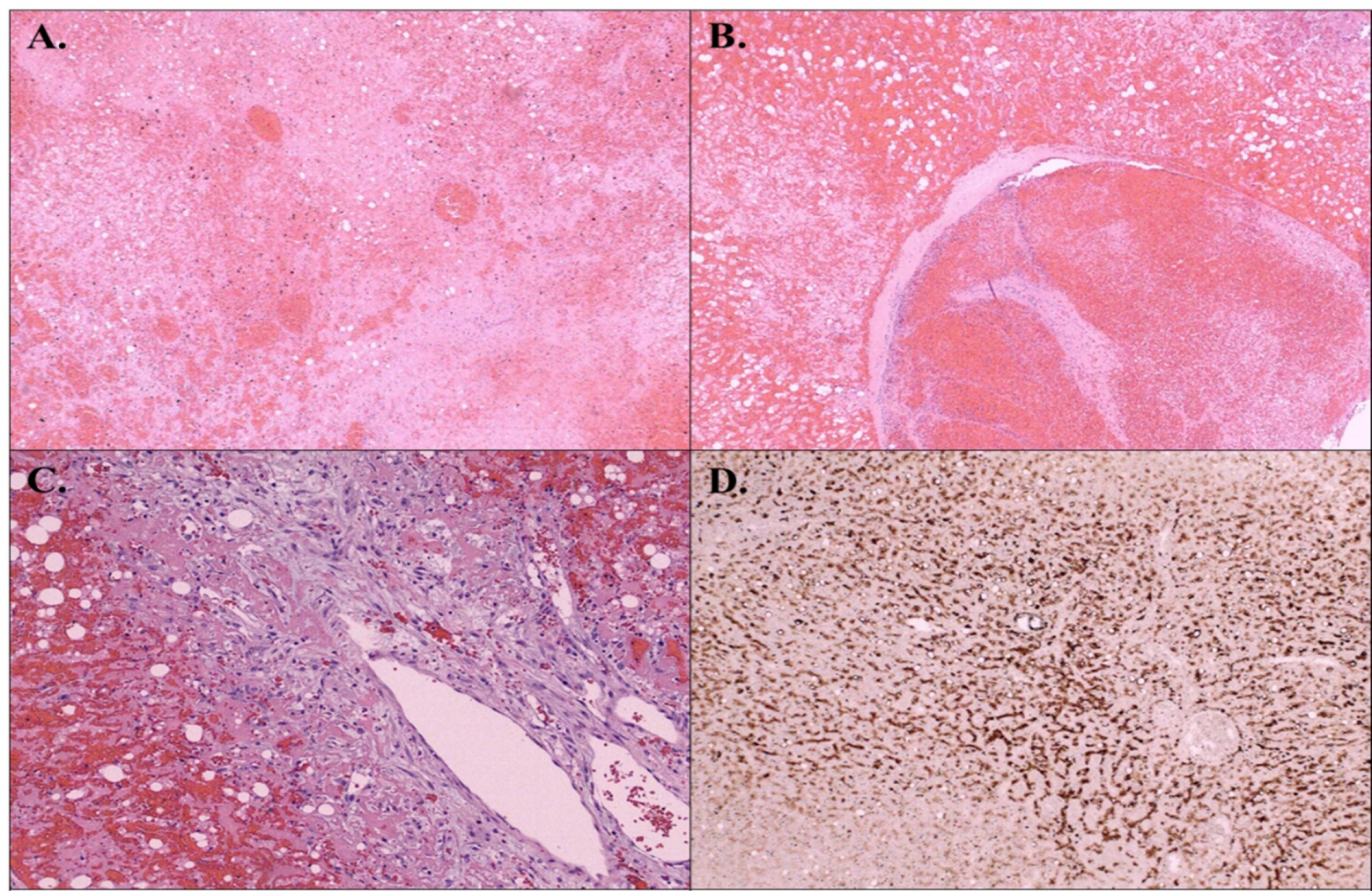
Microscopically, an infarcted, hemorrhagic mass was composed of cords of degenerated epithelioid cells with vacuolated and occasionally lipid-laden cytoplasm (A) A large, dilated vessels with thrombi (B) and papillary endothelial hyperplasia were demonstrated. The mass was surrounded by reactive fibro- and myofibroblastic tissue (C). Epithelioid cells were stained positive with pan-keratin CK AE1/AE3 (D) and negative with CD117. CK: cytokeratin; CD: cluster of differentiation.

Subsequently, the patient underwent an exploratory laparotomy, which revealed free blood with a large amount of blood clots occupying the greater curvature between the stomach and spleen. An infarcted, hemorrhagic, ovoid mass was seen between the fundus of the stomach, diaphragm, and spleen, which was separate from spleen, liver, and greater curvature slightly attached to the diaphragm. Removal of intraabdominal mass was done successfully and followed by biopsies taken from the omentum. Patient had an uncomplicated surgery and remained asymptomatic in the following days. Initially, the lesion was thought to be an accessory spleen on gross examination and the vicinity. The pathological exam of the surgical piece revealed an infarcted, hemorrhagic mass composed of cords of degenerated epithelioid cells with vacuolated and occasionally lipid-laden cytoplasm (Figure 3). Large, dilated vessels with thrombi within some of them and papillary endothelial hyperplasia were noted. Fibro- and myofibroblastic tissue surrounded the mass. Morphologically the epithelioid cells were reminiscent of hepatocytes, and the provided immunohistochemical stains showed them to be diffusely positive for HerPar-1 (hepatocyte origin), cytokeratin (CK) AE1/AE3 and negative for cluster of differentiation (CD)117. There was focal immunoreactivity for CD31 and CD34 between the cell cords. These findings were most consistent with an infarcted inflammatory HCA associated with the reactive papillary endothelial hyperplasia.

## Discussion

The HCA is a rare, benign tumor derived from hepatocytes [1]. The prevalence of these lesions has been rising, given the recent increase in the use of OCP pills and the abdominal imaging modalities [2]. Among the well-known risk factors of HCA are OCP and anabolic steroid use, pregnancy, glycogen storage diseases, and steatohepatitis. OCP use is by far the strongest risk factor, and the dose-dependent association with estrogen prompted the reduction in estrogen dose in the combination OCPs [1-3]. Bleeding (25%), or malignant transformation (5%) can complicate the disease and result in severe abdominal pain [1]. In females, the lesions <5 cm with no associated symptoms or complications can be managed conservatively by terminating the use of OCP, and repeat surveillance imaging. The European guidelines recommend repeat MRI of the abdomen in six months, followed by annual imaging if the tumor is less than 5 cm and is stable in size [4]. The American College of Gastroenterology recommends follow up CT or MRI of the abdomen at six to twelve-month intervals [5]. The duration of monitoring is based on the growth pattern and stability of the lesion over time. Surgical resection is recommended in males due to the higher risk of malignant transformation [1,6,7]. HCA is almost always intracapsular, and to the best of our knowledge, there are only three cases of extracapsular HCA reported in the English literature [8,9]. Our case had a solitary extracapsular HCA complicated with necrosis and intraperitoneal hemorrhage. Another interesting part of this case was the initial presentation with left shoulder pain, which was likely a consequence of phrenic nerve irritation from intraperitoneal bleeding, also known as Kehr’s sign [10]. On initial imaging studies, due to the vicinity and imaging characteristics, the mass was suggested to be originating from the anterior wall of the stomach. Additionally, during surgery, the location of the tumor between the spleen and the stomach resulted in a diagnostic dilemma as the ectopic liver was initially thought to be an accessory spleen.

This work has been already presented as an abstract. (Abstract: Comba I, Henriquez R, Kumar S, Srinivasamurthy R, Wallis-Crespo M, Karasik O, Bhatia L. Extracapsular Hepatocellular Adenoma: A Diagnostic Dilemma. American College of Gastroenterology 2019 Annual Scientific Meetings; October 2019). https://journals.lww.com/ajg/FullText/2019/10001/Extracapsular_Hepatocellular_Adenoma__A_Diagnostic.2470.aspx

## Conclusions

In this report, we aimed to enhance clinicians’ understanding of uncommon presentations of HCA. With recent increase in the prevalence of this condition, especially among patients on OCPs, timely diagnosis and prompt intervention are likely to reduce life-threatening complications.
